# Chlorhexidine Digluconate Effects on Planktonic Growth and Biofilm Formation in Some Field Isolates of Animal Bacterial Pathogens

**DOI:** 10.17795/jjnpp-14298

**Published:** 2014-04-14

**Authors:** Azizollah Ebrahimi, Majid Hemati, Saeed Habibian Dehkordi, Shahab Bahadoran, Sheida Khoshnood, Shahin Khubani, Mahdi Dokht Faraj, Reza Hakimi Alni

**Affiliations:** 1 School of Veterinary Science, Shahrekord University, Shahrekord, IR Iran; 2 Para-Veterinary Sciences, Bu-Ali Sina University, Hamedan, IR Iran

**Keywords:** Biofilms, Chlorhexidine Digluconate, *Salmonella* spp., *Streptococcus agalactiae*, *Escherichia coli*

## Abstract

**Background:**

To study chlorhexidine digluconate disinfectant effects on planktonic growth and biofilm formation in some bacterial field isolates from animals.

**Objectives:**

The current study investigated chlorhexidine digluconate effects on planktonic growth and biofilm formation in some field isolates of veterinary bacterial pathogens.

**Materials and Methods:**

Forty clinical isolates of *Escherichia coli*, *Salmonella* serotypes, *Staphylococcus. aureus* and *Streptococcus* *agalactiae* (10 isolates for each) were examined for chlorhexidine digluconate effects on biofilm formation and planktonic growth using microtiter plates. In all of the examined strains in the presence of chlorhexidine digluconate, biofilm development and planktonic growth were affected at the same concentrations of the disinfectant.

**Results:**

Chlorhexidine digluconate inhibited the planktonic growth of different bacterial species at sub-MICs. But they were able to induce biofilm development of the *E. coli*, *Salmonella* spp., *S. aureus* and *Str. agalactiae* strains.

**Conclusions:**

Bacterial resistance against chlorhexidine is increasing. Sub-MIC doses of chlorhexidine digluconate can stimulate the formation of biofilm strains.

## 1. Background

Biofilms are known to provide a protective environment for pathogenic bacteria, parasites, and viruses aiding their resistance to antimicrobials leading to cause diseases in animals and humans (1). The bacteria inside biofilms have increased resistance to antimicrobial agents (2). The biofilm effect on the bacterial resistance is thought to be related to a direct role of the exopolymeric matrix as a diffusion barrier, to a chemical reaction of some chemicals with the biofilm matrix and to physiological differences between the fixed and suspended organisms (3). Microbial cells in biofilms can easily detach voluntarily or involuntarily from biofilms to aid their dispersal which represents a very important survival strategy (4). Consequently, the bacterial cells which reside in the planktonic phase are thought to be in a phase of moving from one surface to another (5). It is plausible to suggest that these dispersal strategies are therefore the cause of food and water contamination and the animal and human infections/diseases (6, 7). In animal species, the risk of infection is probably considerably greater than the risk in humans. This is due to the difference in animal environment housing. In addition to bacterial ability to growth on body surfaces, biofilms are also able to occupy artificial surfaces including tubing and implants, such as intravenous catheters, teeth and gingiva, lungs, ears, urogenital tract and wounds (8).

The present work investigated whether the biofilm formation in some field strains of animal pathogens can be influenced by chlorhexidine digluconate, at in-use and sub-minimal inhibitory concentrations (sub-MICs). Among cationic antimicrobial agents, biguanides like chlorhexidine digluconate have different behaviours. The biguanides differ from other cationic biocides in such a way that they interact only superficially with the lipid bilayer altering fluidity through cationic displacement and head group bridging (9).

## 2. Objectives

The current study investigated the effect of chlorhexidine digluconate on planktonic growth and biofilm formation in some field isolates of animal bacterial pathogens.

## 3. Materials and Methods

### 3.1. Bacterial Isolates

*Escherichia coli* strains were isolated from the dead poultry referred to veterinary clinic of Shahrekord University by regional poultry farms. *Staphylococcus. aureus* and *Streptococcus* *agalactiae* were isolated from mastitis cow milk in the authors previous works (10, 11).

*Salmonella* serotypes were isolated from, different animals and preserved in the collection of Microbiology Laboratory of Veterinary College. The methods for isolation and identification of all isolates were based on Quinn et al. guidelines (12). Briefly after gram staining, catalase and oxidase examinations, colonies were pure cultured on sheep blood agar plates (Merck, Darmstadt, Germany), hemolysis was scored and then subjected to CAMP (on sheep blood agar), esculin hydrolysis (on TKT) and Rapid hippurate hydrolysis tests in case of gram positives. Also the growth of isolates was examined on MacConkey agar (Merck, Darmstadt, Germany). Carbohydrate utilization was conducted for all isolates in phenol red broth (BBL) with 1% final concentration of the following carbohydrates: lactose, maltose, mannitol, raffinose, salicin and trehalose. Positive reactions were observed by a change from red to yellow after aerobic incubation at 37˚C for 24 hours.

### 3.2. Biofilm Assays

Biofilm formation was evaluated by end-smooth 96-cells micro plates as explained by Tendolkar et al. (13). Briefly, isolates were grown at 37˚C in TSB (Merck, Darmstadt, Germany). The bacterial cells were then pelleted at 6,000 × g for 10 minutes, and the cell pellet was resuspended in 5mL of fresh medium. The optical densities (ODs) of the bacterial suspensions were measured by spectrophotometer (Jenway, OSA, UK) and normalized to an absorbance of 1.00 at 595 nm. The cultures were diluted 1:40 in fresh TSB and 200 μL of cells were dispensed into 12 wells in a single row of a sterile 96-well flat-bottom polystyrene micro titer plate. After incubation at 37˚C for 24 hours, the planktonic cells were aspirated and the wells were washed three times with sterile phosphate-buffered saline (PBS). The plates were inverted and allowed to dry for one hour at room temperature. For biofilms quantification, 200 μL of 0.2% aqueous crystal violet solution was added to each well, and the plates were allowed to stand for 15 minutes. The wells were subsequently washed three times with sterile PBS to wash off the excess crystal violet. Crystal violet bound to the biofilms was extracted with 200 μL of an 80:20 (vol/vol) mixture of ethyl alcohol and acetone, and the absorbance of the extracted crystal violet was measured at 595 nm.

As a control, crystal violet binding to wells was measured for wells exposed only to the medium with no bacteria. All biofilm assays were performed in triplicate, with 12 replicates for each strain per assay. Interpretation of biofilm production was done according to the criteria described by Stepanovic et al. (14). Based on these criteria ODc (optical density cut-off value) is defined as: average OD of negative control + 3 × SD (standard deviation) of negative control, and the biofilms producers are categorized as: no biofilm producer ≤ ODc, weak biofilm producer ODc < ~ ≤ 2 × ODc, moderate biofilm producer 2 × ODc < ~ ≤4 × ODc and strong biofilm producer > 4 × ODc. Where "~" stands for the average of sample ODs. All bacterial isolates were examined for biofilm formation and ten isolates in each genus that were strong biofilm producers were selected to determine the MIC of disinfectant on biofilm formation.

### 3.3. Disinfectant and Determining MICs

Chlorhexidine digluconate was used as the disinfectant in the present study (Sigma-Aldrich, Saint Louis, USA). One percent solution of chlorhexidine digluconate was prepared and preserved for further uses. Standard MICs were determined by broth micro dilution in three separate experiments. Briefly, 50 µL of bacterial suspension (containing 2 × 10^6^ CFU/mL) in Tryptone Soya broth (TSB) medium was added to 50µL of serial two fold dilutions of the disinfectant in TSB in microtiter trays. The plates were incubated for 24 hours at 37˚C and checked for turbidity. The MIC was defined as the lowest concentration of disinfectant inhibiting zone of the bacterial growth.

### 3.4. The Test of Disinfectant Effect on Planktonic Growth and Biofilm Formation

To consider the effect of disinfectant on biofilm formation Houari and Martino`s method was used (15). The method had the following steps:

- Turbidity of the examined bacteria fresh culture was adjusted to 0.5 McFarland.
- 50 µL of the above bacteria and the same amount of different dilutions of disinfectant were mixed (dilutions of 2 MIC, MIC, 1/2 MIC, 1/4 MIC, 1/8 MIC, 1/16 MIC and 1/32 MIC).
- Microplates were incubated in 37˚C for 24 hours.
- Absorbance of planktonic growth of bacteria in 630nm was determined using ELISA microplate reader (Bio-Tek, Winooski, USA).

### 3.5. Statistical Analysis

The results were analyzed and compared using Duncan multi range tests at probability level of %5.

## 4. Results

### 4.1. Properties of Strains

The most efficient biofilm formation was observed with the *S. aureus* and *Str. agalactiae* strains and the least two with the *E. coli* and *Salmonella* serotypes. For each strain, the MIC was determined by the conventional twofold dilution method in TSB and were 0.0009, 0.0009, 0.002 and 0.002 (% w. v) for *E. coli*, *Salmonella* serotypes, *S. aureus* and *Str. agalactiae* respectively. *Staphylococcus. aureus* and *Str. agalactiae* were the least sensitive strains, and *Salmonella* spp. was the most sensitive strain to chlorhexidine digluconate.

### 4.2. Effects of Chlorhexidine Digluconate on Planktonik Bacterial and Biofilm Growth

**Figure 1.**
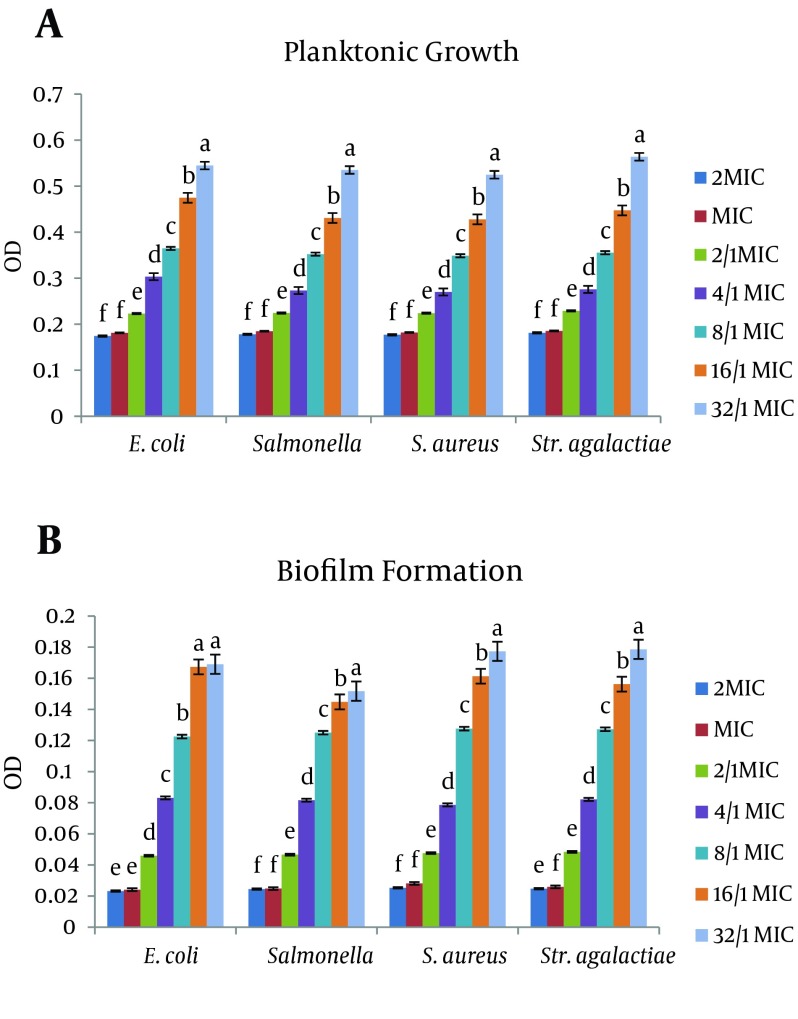
Effect of Chlorhexidine Digluconate on Planktonic Growth and Biofilm Formation Plates were inoculated with the bacterial suspensions (turbidity of 0.5 MacFarland) with the chlorhexidine at different concentrations and incubated at 37˚C for 24 hours, OD = 630 nm. Data are expressed as the mean of three separate experiments in triplicate (± SD), * P < 0.05. * Indicates statistically significant difference from the previous column.

In Figure 1, the biofilm formation and planktonic growth of the four strains in the presence of different concentrations of chlorhexidine are presented. For all the strains, biofilm development and planktonic growth were affected at the same concentration of disinfectant. The average Planktonic growth of strains after MIC concentration was significant in all bacteria. 

This result was also true in the case of biofilm formation (except for *E. coli* bacteria at 1.32 MIC concentrations). The maximum OD of Planktonic growth was in *E. coli*, *Salmonella* spp., *S. aureus*, *Str. agalactiae* as 0.39, 0.39, 0.5, and 0.53, respectively. Also the highest OD related to biofilm formation was 0.182, 0.177, 0.2, and 0.2, respectively.

Interactions between disinfectants and bacteria in the cases of *E. coli*, *Salmonella* spp. and *S. aureus* strains were significant at the planktonic growth stage, and *Salmonella* spp. on the biofilm formation stage. In such a way that the highest rate of planktonic growth compared to MIC concentration in E.coli, *Salmonella* spp., *S. aureus* were respectively, 53.3%, 54.7%, and 53.7% in 1.32 MIC. The lowest growth rates were respectively 8.3%, 5.1%, and 2.9%, in 1.2 MIC concentrations. The highest and the lowest growth biofilm formation rates compared with MIC concentration in *Salmonella* spp. were respectively 84.8% in 1.32 MIC concentration and 31.2% in 1.2 MIC concentration (P < 0.05).

## 5. Discussion

The present study was designed to determine the inhibiting effect of chlorhexidine on biofilm and planktonic growth of some animal bacterial pathogens. No significant planktonic growth and biofilm formation were observed in the presence of chlorhexidine in concentrations of one and two fold MIC, P < 0.05. It can imply that chlorhexidine concentrations higher than MIC have similar effects on planktonic growth and biofilm formation and there is no need to use concentrations higher that MIC to control bacterial infection. Of course, the law is gradually void in cases including the presence of resistance genes, mutation (16, 17) and resistance acquiring (16, 18). In addition, organic materials, pH, temperature, water rigidity, chemical harnesses and contact time are involved in the effect of disinfectants (19-21).

Although planktonic growth chart, indicated the growth of strains (due to average of strains growth) at lower concentrations of MIC but this increase in 1.2 MIC concentration and in some cases in 1.4 MIC was not visible with the naked eye. Therefore, the inhibitory effect of chlorhexidine on Planktonic growth can be acceptable. In the stages of biofilm formation, no significant interaction was observed between antiseptic and antibacterial components in *E. coli*, *Str. agalactiae* and *S. aureus*. However, the biofilm formation growth was significant, with the decrease of disinfectant concentration, in all samples (except *E. coli* in 1.32 MIC). Due to the release of uptake dye by bacteria, this increase was clearly evident and will be more visible by MIC reduction; this reflects the inability of chlorhexidine to prevent bacterial biofilm formation. Comparison of the highest and the lowest increase rates of planktonic growth and formation of *Salmonella* spp. biofilm confirmed this result. In fact, results showed that sub-MIC doses of chlorhexidine digluconate can stimulate the strains biofilm formation. This phenomenon can have deleterious effects because biofilm formation is thought to play an important role in the survival of virulent strains of these bacteria (22). *S. aureus* has been reported to be a concern in postoperative wound biofilm infections (23) and mastitis (24). Cross infection of MRSA between animals and humans has been recognised (25). The evidences show that biofilm life manner cause resistance increase against anti microbial products. In fact, one of the bacterial resistance methods is biofilm growth where the cells survive generally because of disinfectants inability to reach cells, which will cause bacteria sensivity reduction (26). The cationic anti microbial mode of action against bacterial cells involves a general perturbation of lipid bilayer membranes (27). Low concentrations of chlorhexidine digluconate bind firmly to exposed anionic sites on the cell membranes. Such interactions have previously proved to decrease membrane fluidity , to affect the osmoregulatory and physiological functions of the cell membranes (28), and also biofilm development.

At higher, in-use concentrations, the interactions are more severe and cause the membrane to lose its structural integrity and allow leakage of cellular materials (9). The stimulation of *S. aureus* and *Str. agalactiae* biofilm formation by chlorhexidine digluconate seems to be unrelated to an effect on bacterial growth of planktonic cells, but effects on cell viability cannot be ignored. Thus, the presence of a biocide at low concentration could decrease planktonic viability and protect against planktonic growth. In conclusion, chlorhexidine was able to inhibit biofilm formation of different bacterial species at conventional in-use concentrations. Nevertheless, the biofilm formation induction observed in the *Salmonella* spp. strains in the presence of sub-MIC of disinfectant raises concern over the inappropriate use of cationic disinfectants. Given the prevalence of biofilms in natural environments, it is not surprising that these growth forms are responsible for infection in humans and animals.
